# Corrections of BDS Code Multipath Error in Geostationary Orbit Satellite and Their Application in Precise Data Processing

**DOI:** 10.3390/s19122737

**Published:** 2019-06-18

**Authors:** Weiwei Song, Qiong Wu, Xiaopeng Gong, Fu Zheng, Yidong Lou

**Affiliations:** 1 GNSS Research Center, Wuhan University, Luoyu Road 129, Wuhan 430079, China; SWW@whu.edu.cn (W.S.); wqiong_v@whu.edu.cn (Q.W.); ydlou@whu.edu.cn (Y.L.); 2 School of Electronic and Information Engineering, Beihang University, 37 Xueyuan Road, Beijing 100083, China; fzheng@buaa.edu.cn

**Keywords:** geostationary orbit satellite, multipath error, wavelet transform, satellite clock estimation, ionospheric delay

## Abstract

Multipath error is a main error source in Global Navigation Satellite System (GNSS) data processing, which cannot be removed by a differential technique because of the strong relationship with the environment around the station. The multipath effect of the code observables is more complex than that of the carrier-phase observables, especially for BeiDou Navigation Satellite System (BDS) geostationary orbit (GEO) satellites. In this contribution, we deeply analyzed the characteristic and effect on the precise data processing of GEO satellite multipath errors based on a large number of permanent GNSS stations. A linear combination of code and carrier-phase observables was used to analyze the characteristics of repeatability for BDS GEO’s multipath. Then, a correction method was proposed to eliminate the multipath error of the GEO code observables, based on wavelet transform. The experiment data were collected at 83 globally distributed stations, from multi-GNSS experiments and national BDS augmentation systems, from days 32 to 66 in 2017. The results show that the systematic multipath variation component of the GEO code observables can be obtained with wavelet transform, which can significantly contribute to correcting the multipath error of GEO satellites. The average root mean square error (RMSE) of the multipath series is decreased by approximately 19.5%, 20.2%, and 7.5% for B1, B2, and B3, respectively. In addition, some experiments, including ionospheric delay extraction and satellite clock estimation, were conducted in simulated real-time mode in order to validate the effect of the correction methods. For the ionospheric delay estimation, the average RMSE of the slant ionospheric delay is reduced by approximately 15.5%. Moreover, the multipath correction can contribute greatly to shortening the convergence time of the satellite clock estimation of the BDS GEO satellites.

## 1. Introduction

Multipath is a major error in Global Navigation Satellite System (GNSS) error sources. As multipath is associated with the site environment and cannot be cancelled out by differential technique, it will exert considerable effects on the quality of GNSS signals, and should be well-treated in high-precision GNSS data processing. For example, severe multipath effects will slow down the convergence of precise point positioning (PPP) and decrease the reliability of PPP ambiguity resolution [1,2]. As for its positioning in a dynamic environment, sometimes, a poor geometry results in the positioning accuracy being more sensitive to multipath errors [3,4,5]. As for the ionospheric delay estimation, the geometry-free combinations using phase smoothing code are widely used to reduce noise, and avoids directly resolving ambiguities [6]. However, the multipath error cannot be cancelled out when averaging over a continuous arc, and thus it affects the precision of the ionospheric delay estimation [7,8]. Furthermore, in real-time GNSS clock estimation, phase and range observables contribute to clock solutions in different ways [9]. The highly precise phase observables determine only the accurate temporal change of clock biases, whereas the pseudo-range observables determine best the constant offset of each clock [10]. The unmodeled error in pseudo-range will surely increase the convergence time of the initial clock bias estimation, or even introduce an initial clock bias error [11].

Different methods have been proposed to eliminate or reduce the effects of multipath error; these methods can mainly be divided into wavelet transform, signal-to-noise ratio (SNR), and sidereal filtering. Among them, wavelet transform is proposed to separate the double-difference observables of the Global Positioning System (GPS) into low-frequency deviations and high-frequency noise [12]. On the basis of this method, the multipath errors in GPS-phase observables are extracted and used to correct the original observables [13,14]. To improve the robustness of the method, the authors of [15] proposed stationary wavelet transform on the basis of wavelet transform, to extract multipath effects from double-difference residuals of the GPS code range observables. Different from the wavelet transform method, a method named SNR, which can reflect the effect of multipath errors on signals, has been proposed [16,17]. The authors of [18] verified the capability of the SNR method to detect multipath interference with triple-frequency observables. The results tested by GPS BLOCK IIF satellite observables showed that the process is also sensitive to diffraction in a low-multipath environment. Studies on the application of sidereal filtering have also been conducted [19]. The authors of [20] recalculated the orbital repetition period of the GPS satellites, and applied it to filter the 1-Hz GPS position estimation by carefully considering the ground track repeat of the GPS satellites. Moreover, a new sidereal filtering algorithm based on single-difference was proposed so as to obtain the multipath correction of each satellite. The experiments demonstrated that the positioning accuracy could be improved by 7% compared with that of the double-difference method [21].

The aforementioned studies have mainly focused on the multipath error mitigation of GPS satellites. The BeiDou Satellite Navigation System (BDS) adopts heterogeneous constellations; thus, the characteristics of the multipath errors of different satellite types need further investigation. Severe multipath errors were found to exist in the code observables of BDS geostationary orbit (GEO) satellites for all of the triple-frequency observables [22,23,24,25,26,27]. Furthermore, it was demonstrated that the multipath of the GEO satellites mainly presented low-frequency characteristics, and its repetition period was almost consistent with the orbital period of the satellites [24,27]. It has been validated that the multipath of BDS GEO satellites constraints the high-precision positioning service of BDS, which cannot be ignored [22,25]. In order to correct the multipath error, different methods such as the Code Noise and Multipath Correction technique, wavelet transform method, and adaptive wavelet transform method were adopted to remove the low-frequency component of the multipath errors. Furthermore, the experiments of the single point positioning (SPP) and long-baseline static positioning were used to validate the effectiveness of the multipath corrections. The results show that the accuracy of the SPP and long-baseline static positioning could be greatly improved after multipath corrections were applied [22,23,26,27]. 

The above research mainly focused on the analysis of the multipath characteristics and its application in precise positioning. The influence of the multipath error on the precise data processing, such as the ionospheric delay extraction and satellite clock estimation, still need further investigation. Thus, the present work firstly analyses the multipath characteristics of BDS GEO satellites, based on a large number of permanent GNSS stations. On the basis of wavelet transform, a periodic correction method is proposed to eliminate the multipath error of every station. Then, the multipath correction method of the GEO satellites was applied to the BDS high-precision data processing in simulated real-time mode, including ionospheric delay extraction and satellite clock estimation, so as to verify the influences of GEO’s multipath error on BDS high-precision data processing.

## 2. Mathematical Models

In this section, the basic mathematical models, including the multipath combination method and wavelet transform method, are briefly introduced.

### 2.1. Multipath Combination

Assuming that the code rang and the carrier phase observables of the receiver (*r*)*,* to the visible satellite (*s*) at a certain frequency (*f_i_*) are $P_{r,i}^{s}$ and $\Phi_{r,i}^{s}$, which can be expressed by the following equation:

(1) $$\begin{array}{c} P_{r,i}^{s}=\rho_{r}^{s}+t_{r,sys}-t^{s}+b_{r,i}-b_{i}^{s}+trop_{r}^{s}+iono_{r,i}^{s}+\varepsilon_{P}\\ \Phi_{r,i}^{s}=\rho_{r}^{s}+t_{r,sys}-t^{s}+trop_{r}^{s}-iono_{r,i}^{s}-\lambda_{i}\cdot N_{r,i}^{s}+\varepsilon_{\Phi } \end{array}$$

where $\rho_{r}^{s}$ denotes the geometric distance from the station to the satellite;$\;t_{r,sys}$ denotes the receiver clock error associated with GNSS; $t^{s}$ denotes the satellite clock error;$\;b_{r,i}$ and $b_{i}^{s}$ denote the hardware delay deviation of the code rang observables in frequency (*f_i_*) for the receiver and satellite, respectively;$\;trop_{r}^{s}$ and $iono_{r,i}^{s}$ denote the tropospheric delay and ionospheric delay on the signal propagation path, respectively;$\;N_{r,i}^{s}$ is the float ambiguity, consisting of integer ambiguity and initial phase bias; $\lambda_{i}$ is the phase wavelength; and $\varepsilon_{P}$ and $\varepsilon_{\Phi }$ denote the noise on the code range and carrier phase observables, respectively. The multipath combination is a linear combination of single-frequency code range and dual-frequency carrier-phase observables [28], which can be described as follows:

(2) $$C_{r,i}^{s}=P_{r,i}^{s}-\frac{f_{i}^{2}+f_{j}^{2}}{f_{i}^{2}-f_{j}^{2}}\Phi_{r,i}^{s}+\frac{2f_{j}^{2}}{f_{i}^{2}-f_{j}^{2}}\Phi_{r,j}^{s}$$

where $C_{r,f_{i}}^{s}$ is a multipath combination; i and j denote different frequencies of *f_i_* and *f_j_*, respectively; and *i* ≠ *j*; The multipath combination derived by Equation (2) contains not only the multipath, but also the ambiguity, hardware delay, and noise. When no cycle slip occurs, the ambiguity is assumed to be a constant value, and can be derived through averaging over the epochs. Thus, a zero-mean term for multipath effect on frequency *f_i_* can be derived as follows:

(3) $$MP_{r,i}^{s}=C_{r,i}^{s}-{\bar{C}}_{r,i}^{s}$$

where $MP_{r,i}^{s}$ is a zero-mean term multipath combination, and ${\bar{C}}_{r,i}^{s}$ is the average value of $C_{r,i}^{s}$ over time. Theoretically, multipath on carrier-phase observables will not exceed one quarter of the carrier wave cycle, which is approximately several centimeter’s [18]. In comparison with the multipath on code observables, the multipath on carrier-phase observables can be neglected. Thus, multipath combination can be used for code multipath analysis. However, the multipath series derived through Equation (3) consists of low-frequency and high-frequency components, which should be separated in order to analyze the characteristic of multipath errors.

### 2.2. Wavelet Transform

Wavelet transform is used to represent or approach a signal with a family of wavelet functions generated from a prototype function. It has been widely used in GNSS data processing [14,21,29,30]. According to research, GPS bias terms, such as multipath and ionospheric delay, behave like low-frequency noise, and the observation noise behaves as high-frequency noise. Hence, the GPS bias terms are concentrated in the narrow low-frequency band, and a high-frequency resolution is needed in order to identify them [30]. Multi-resolution analysis can provide a formal approach to constructing the wavelet basis. The basic concept of multi-resolution analysis is to analyze the signal at different scales by using filters of different cut-off frequencies. The signal is passed through a series of high-pass filters (HPF) in order to analyze the high frequencies, and it is passed through a series of low-pass filters (LPF) in order to analyze the low frequencies. Thus, wavelet transform can be used to achieve enough frequency resolution to discriminate the multipath error in the original GNSS observables [13]. 

Figure 1 illustrates the multi-resolution analysis process using wavelet transform. Applying a narrow daughter wavelet to the original signal is equivalent to applying a HPF, which completes path 1. Extracting the leading low-frequency requires applying a number of daughter wavelets that are wider than the signal you need to match, then applying a final daughter wavelet that becomes a HPF completing path 2. Moreover, the low-frequency components obtained by the decomposition can be used for signal reconstruction, and detailed information can be found in the literature [21]. As for the details of the wavelet transform, they are listed in the Appendix A.

The key issue of wavelet transform is the determination of the wavelet function and decomposition level, but there is no unified theoretical standard so far. The Daubechies wavelets are a good option, as they are orthonormal bases of compactly supported wavelets [31]. We did not carry out any specific theoretical analysis on the selection of wavelet basis functions, but directly used the Daubechies wavelet on the basis of a previous study [32]. The decomposition level was determined to be three for data processing.

## 3. Experimental Analysis

Given that the multipath error is related to the station environment, if the station environment remains the same, then the difference in multipath error among continuous days will be extremely small. Therefore, the multipath error can be extracted from the historical data, and can be applied to the subsequent data processing. In this section, 83 globally distributed stations were used for the multipath characteristic analysis of BDS GEO satellites. Some of these stations were used for their ionospheric delay extraction and satellite clock estimation in simulated real-time mode in order to verify the effectiveness of the proposed method.

### 3.1. Data Collection and Processing Strategy

A GNSS network with 83 stations, which were from the multi-GNSS experiment campaign (MGEX) of the international GNSS service (IGS) and the National BDS Augmentation Service System (NBASS), was used in this paper [33,34,35]. Among them, the data of MGEX was achieved through the Crustal Dynamics Data Information System (CDDIS) [36]. The station distribution is shown in Figure 2. Some stations could not track the GEO satellites, which are mostly located in the area of America. Only the observables from 78 stations could be used for GEO satellite analysis. The data were collected from days 32 to 66 of 2017 with 30 s intervals, and the corresponding calendar dates were from 1 February 2017 to 7 March 2017.

The multipath combination consists of multipath errors and noises of observables, which can be divided into low-frequency and high-frequency components. These random noises have a low or no correlation between different days. Therefore, the two parts must be separated, and the multipath error must be extracted from the original multipath series. 

### 3.2. GEO Satellite Multipath Calibration and Elimination

In this section, the characteristics of the GEO satellite multipath on triple-frequency observables are analyzed and discussed. Then, the low-frequency part of the multipath series is extracted and applied to correct multipath error. 

#### 3.2.1. Multipath Calibration

BDS GEO satellites can provide triple-frequency code observables. Thus, an analysis is performed to illustrate whether the multipath errors at different frequencies are correlated. Figure 3 presents the one-week multipath time series on a triple-frequency of BDS GEO satellite (C04) at station CUT0. To understand the characteristics of the multipath series more clearly, the multipath series at triple-frequency of date-of-year (DOY) 37 (6 February 2017) were also amplified separately. For simplicity, multipath combinations on the frequency B1, B2 and B3 are abbreviated to MP1, MP2 and MP3, respectively. As is shown in Figure 3, the amplitude fluctuations of the multipath series vary with the frequency. For example, the amplitude of MP1 series is about 2.0 m, which is significantly larger than those of MP1 and MP3. Overall, the multipath series of all of the triple-frequencies shows evident periodic repeatability. The repetition period is approximately one day, which is close to the GEO satellite orbit period. On the basis of the above analysis, the periodical features might be used to eliminate the multipath effects and to improve the quality of the precise data processing.

The method of wavelet decomposition and reconstruction proposed is applied so as to derive the low-frequency component of the multipath series. With the wavelet decomposition and reconstruction, the low-frequency and high-frequency components in the multipath time series for each frequency can be separated. Figure 4 shows the three-level Daubechies wavelet decomposition of the multipath series. As can be seen from Figure 4, the extrema and amplitudes of the noise decrease with the increase of the decomposition level. Moreover, the low-frequency component extracted form the third level is extremely close to the original multipath series. So, it is also called the approximation. Thus, the low-frequency components are expected to be used for correcting the original observables of both the current and subsequent periods.

Figure 5 presents the correction result of the multipath series for the BDS GEO satellite (C04) at CUT0. The low-frequency component is extracted from the multipath series and then applied to correct the multipath error. From the figure, we know that the multipath error is greatly decreased when the low-frequency component is removed from the multipath series. It is clearly seen that the variation amplitudes of the MP1, MP2, and MP3 series decrease from 2.0 m to 1.0 m, from 1.0 m to 0.5 m, and from 1.0 m to 0.3 m, respectively. Generally, after the low-frequency multipath errors are subtracted, the most systematic variations in the original observables are eliminated, and the residual series becomes close to white noise. The results demonstrate that the low-frequency component derived by the wavelet analysis can be used to eliminate the effect of multipath error.

#### 3.2.2. Multipath Elimination

According to Section 3.2.1, the low-frequency component of the multipath series derived by the wavelet transform can greatly decrease the effect of the multipath error. To evaluate the improvement due to the low-frequency removal, the root mean square error (RMSE) of the one-week multipath series was computed for both without and with the multipath correction, respectively. 

Figure 6 shows the RMSE of the GEO satellite (C04) at station CUT0. The blue bars represent the RMSE without the multipath error correction, while the red bars represent the RMSE with the multipath error correction. It is obvious that the RMSE of the multipath series considerably decreases with the low-frequency components of the previous day, being removed from the raw time series. The RMSE of the multipath series at station CUT0 on the frequencies of B1, B2, and B3 is decreased by 42.0%, 34.0%, and 60.0% according to the average RMSE, respectively. The RMSE of MP3 is smaller than that of MP1 and MP2, mainly because of its low noise of observable.

Table 1 shows the statistical results of the multipath series without and with the multipath correction for each GEO satellite from all of the stations. With the multipath correction, the accuracy of the multipath series at three frequencies is improved. For example, the RMSE of C01 satellite is decreased from 0.24, 0.21, and 0.25 m to 0.20, 0.17, and 0.23 m for MP1, MP2, and MP3, respectively. Finally, the average RMSE of the multipath series at the three frequencies was decreased by approximately 19.5%, 20.2%, and 7.5%, respectively. As the multipath is related to the environment around the station, the multipath effects differ among the different stations, which results in the difference between Figure 6 and Table 1.

Considering that the performance of the multipath correction is related to the time delay from the corrected day, we deeply evaluated the effect of the multipath elimination by using corrections from different time delays. The low-frequency components were extracted from the multipath series with time delays, from one-day to seven-days. Figure 7 shows the statistical results of the C04 satellite in frequencies B1 and B2, and the vertical axis shows the improvement with the multipath correction. From the figure, it is clear that with the time delay increase from one-day to seven-days, the improvement of the multipath RMSE gradually decreases. For example, the best results were obtained using the low-frequency component extracted from the previous day (delay time of one-day). The largest improvement could reach up to 0.3 m, and the average improvements were about 0.05 m and 0.04 m for MP1 and MP2, respectively. However, the results obtained by the corrections from seven-days ago were the worst. The multipath error become even worse for some stations when the corrections were derived from several days ago. This is because with the time extension increase, the correlation of the multipath series between the day correction derived and the day correction applied was decreased. Thus, to eliminate the multipath error as much as possible, we suggest that the latest observable is used to derive the multipath correction.

### 3.3. Validation of BDS Precise Data Processing

From Section 3.2, it is validated that the low-frequency component extracted from the multipath series can greatly eliminate the multipath error, and the amount of improvement will decrease with the increase in time span between the day correction derived and the day correction applied. In this section, several experiments were used to validate the performance of the multipath correction, such as the ionospheric delay extraction and satellites clock estimation. All of these experiments were performed in simulated real-time mode, and the time span was chosen as one day. Moreover, they were all performed with two schemes, that is, using the original observables without and with the multipath correction. 

#### 3.3.1. Ionospheric Delay Extraction

A geometry-free combination eliminates the geometry-related errors, which are widely used in ionospheric delay estimation [37], and can be described as follows:

(4) $$\{\begin{array}{c} P_{r,GF}^{s}=P_{r,i}^{s}-P_{r,j}^{s}=b_{r,i}-b_{r,j}-\left(b_{i}^{s}-b_{j}^{s}\right)+iono_{r,i}^{s}-iono_{r,j}^{s}+\varepsilon_{P,GF}\\ \Phi_{r,GF}^{S}=\Phi_{r,i}^{s}-\Phi_{r,j}^{s}=-(iono_{r,i}^{s}-iono_{r,j}^{s})-\left(\lambda_{i}\cdot N_{r,i}^{s}-\lambda_{j}\cdot N_{r,j}^{s}\right)+\varepsilon_{\Phi ,GF} \end{array}$$

where $\varepsilon_{P,GF}$ and $\varepsilon_{\Phi ,GF}$ denote the noise corresponding to the observables’ geometry-free combination of code and phase, respectively. The hardware delay was generally treated as a constant value during a period. When there was no cycle jump in the observables, the combination of ambiguity was also a constant. Ignoring the influence of the high-order terms of the ionospheric delay, the hardware delay and ambiguity can be obtained by $P_{r,GF}^{s}$ and $\Phi_{r,GF}^{S}$, as shown in the following equation.

(5) $$\langle P_{r,GF}^{s}+\Phi_{r,GF}^{s}\rangle \;=\;b_{r,i}-b_{r,j}-\left(b_{i}^{s}-b_{j}^{s}\right)-\left(\lambda_{1}\cdot N_{r,1}^{s}-\lambda_{1}\cdot N_{r,2}^{s}\right)$$

where 〈·〉 is an average operator for multi-epochs to eliminate noises. The ionospheric delay of the signal propagation path can be obtained by using the observables of the continuous arc through the phase smoothing code range.

(6) $$iono_{r,i}^{s}-iono_{r,j}^{s}=\left[\langle P_{r,GF}^{s}+\Phi_{r,GF}^{s}\rangle -\Phi_{r,GF}^{s}-\left(b_{r,i}-b_{r,j}-\left(b_{i}^{s}-b_{j}^{s}\right)\right)\right]$$

where $b_{r,i}-b_{r,j}$ and $b_{i}^{s}-b_{j}^{s}$ are differential hardware delays, which are also named differential code biases (DCBs). The satellite and receiver DCBs can be corrected by the products provided by IGS and averaged through different satellites, respectively. 

According to the above description, the accuracy of the code observables has an impact on the accuracy of the slant path ionospheric delay extraction. The observables of 54 stations within the latitude range of W60° to E145° were used in estimating the ionospheric delay in order to study the influences of the multipath error on ionospheric delay extraction, and the observable period is from DOY 46 to 66 in 2017 (16 February 2017 to 7 March 2017). The ionospheric delay estimation is also performed by using the two schemes (i.e., without and with multipath correction). In addition, as the accuracy of the ionospheric delays calculated by PPP mainly depend on phase observables, they are used as the true values in order to evaluate the accuracy of the ionospheric delay obtained by geometry-free combination.

Figure 8 shows the time series of the ionospheric residuals on the slant path at station CUT0 without and with the correction of multipath errors. From the figure, it is clear that the accuracy of the ionospheric delay is considerably improved by correcting the multipath error. For example, without the multipath correction, the ionospheric residuals may reach up to eight total electron content unit (TECU) at the initial time of processing. However, with the multipath correction, the ionospheric delay residuals were only approximately three TECU at the initial time. This is because the error of code observables mainly contains white noise, which can be decreased with multi-epochs observables after the low-frequency component of the multipath is removed. Finally, the RMSE of the ionospheric residuals in the first 12-hours of station CUT0 are decreased from 2.54 TECU to 1.46 TECU, which is improved by approximately 42.4%. 

Table 2 presents the RMSE statistics of the ionospheric residual for each GEO satellite. From the table, with correcting the multipath error, the accuracy of the extracted ionospheric delay is improved. The improvements of C01 to C05 are 14.1%, 11.3%, 15.7%, 17.4%, and 18.7%, respectively.

#### 3.3.2. Satellite Clock Estimation

The observables of the 83 stations in the period of DOY 33 to 47 in 2017 (2 February 2017 to 16 February 2017) were used for the BDS satellite clock estimation in order to verify the effect of correcting the GEO’s multipath error. The BDS satellite clock was estimated in the simulated real-time mode, and they were set as re-initialization for each day. For the other details of the processing strategy, we refer to the study of [38]. Finally, the multi-system satellite clocks final products provided by Deutsches GeoForschungsZentrum (GFZ) were used to evaluate the accuracy of the satellite clocks’ estimation [39]. 

Figure 9 shows the time series of the BDS satellite clock errors without and with multipath correction. As we were concerned about the convergence time of the satellite clock, the biases of satellite clock between the two products were removed. As shown in Figure 9, with the multipath correction being used, the accuracy of the satellite clock was considerably improved at the first several hours. For example, when the convergence time was approximately two hours, the satellite clock errors of C02 were approximately 0.21 ns and 0.12 ns for without and with multipath correction, respectively. For the satellite clock estimation without multipath correction, 1.5, 2.2, and 3.1 hours were needed in order to converge to 0.2 ns for the C01, C02, and C03 satellites, respectively; however, for the satellite clock estimation with multipath correction, it only needed 0.81, 1.32, and 0.20 hours, respectively. The convergence time was shortened by 46.0%, 40.0%, and 93.5% for the C01, C02, and C03 satellites, respectively. Since C04 and C05 satellites are located in the westernmost and easternmost parts respectively; hence, the convergence time of their satellite clocks was evidently larger than that of the C01, C02, and C03 satellites. Thus, the improvements of the C04 and C05 satellite clock were not evident when the threshold is 0.2 ns. When the threshold was set as 0.5 ns, the convergence times of the C04 and C05 satellite clock were shortened by about 17.6% and 20.2%, respectively.

## 4. Conclusions

The multipath errors cannot be eliminated by the differential technique, because they are closely related to the environment around the station. The existence of multipath errors will greatly decrease the accuracy of the GNSS data processing, especially for the BDS GEO satellites. To correct the multipath error and improve the accuracy of the BDS precise data processing, we deeply investigated the multipath characteristics of the BDS GEO satellites by using multipath combinations. Given that the multipath combinations consist of noises and low-frequency multipath errors, wavelet transform is used to derive the low-frequency components of the multipath errors. Then, the low-frequency components of the multipath from the first day can be used to correct the multipath errors in real-time.

To validate the method proposed, the multipath series was initially analyzed. The results show that the multipath series was close to white noise when multipath correction was used. The average RMSE of the multipath series was decreased by approximately 19.5%, 20.2%, and 7.5% for B1, B2, and B3, respectively. However, the improvements decreased with the increase in span time delay. For example, the RMSE of the multipath will even become worse for some stations when the time delay is seven-days. Thus, we suggest that the multipath correction should be derived by the latest observables. Moreover, BDS precise data processing, including ionospheric delay extraction and satellite clock estimation, is conducted in simulated real-time mode in order to validate the effectiveness of the multipath correction. For the ionospheric delay extraction, the ionospheric delay calculated by PPP was used as the reference to evaluate the accuracy of the ionospheric delay estimated without and with multipath correction. The results show that the average RMSE of the ionospheric delay on the slant path is decreased by 15.5% with eliminating the multipath error. As for the satellite clock estimation, the results demonstrate that the multipath correction can greatly shorten the convergence time. For example, the times for the satellite clocks were shortened by 46.0%, 40.0%, and 93.5% to converge to 0.2 ns for C01, C02, and C03, respectively. Furthermore, they were shortened by 17.6% and 20.2% to converge to 0.5 ns for the C04 and C05 satellites, respectively. All of these results show that the proposed method can be used to correct multipath error at permanent GNSS stations, which is helpful for real-time BDS precise data processing, such as ionospheric delay estimation and satellite clock estimation. It is also expected that with the multipath error of the IGSO/MEO satellites being investigated, it will improve the accuracy of the BDS precise data processing further.

## Figures and Tables

**Figure 1:**
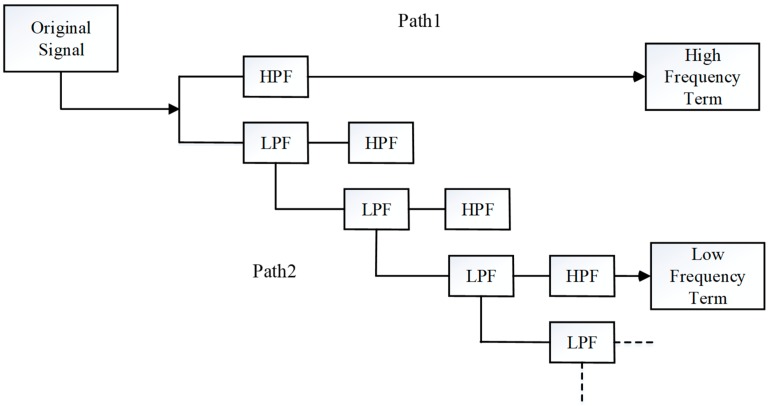
Multi-resolution analysis using wavelet transform [30].

**Figure 2:**
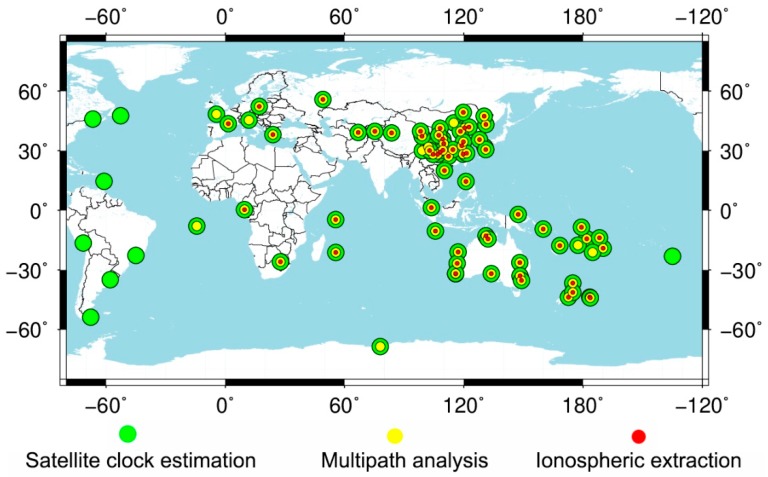
Distribution of the Global Navigation Satellite System (GNSS) stations from the multi-GNSS experiment campaign (MGEX) of the international GNSS service (IGS), and the National BDS Augmentation Service System (NBASS). The points in green, yellow, and red represent the stations used for the satellite clock estimation, multipath analysis, and ionospheric delay extraction, respectively.

**Figure 3:**
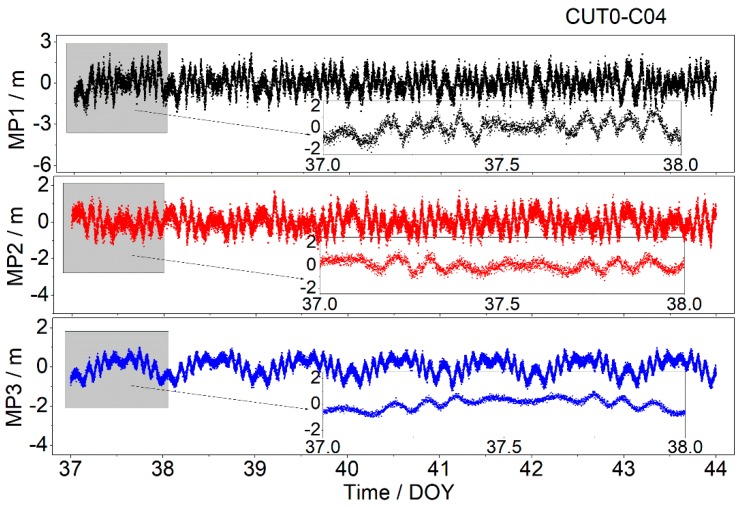
Multipath series of MP1 (top), MP2 (middle), and MP3 (bottom) for C04 at CUT0 station from date-of-year (DOY) 37 to 44 in 2017.

**Figure 4:**
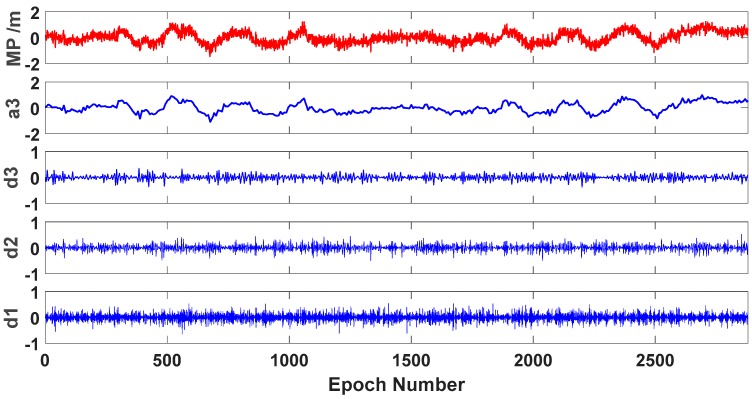
Wavelet decomposition and reconstruction of MP2 at CUT0 station (C04); MP represents the original multipath series; a3 represents the low frequency component of the third level; d1–d3 represent the details.

**Figure 5:**
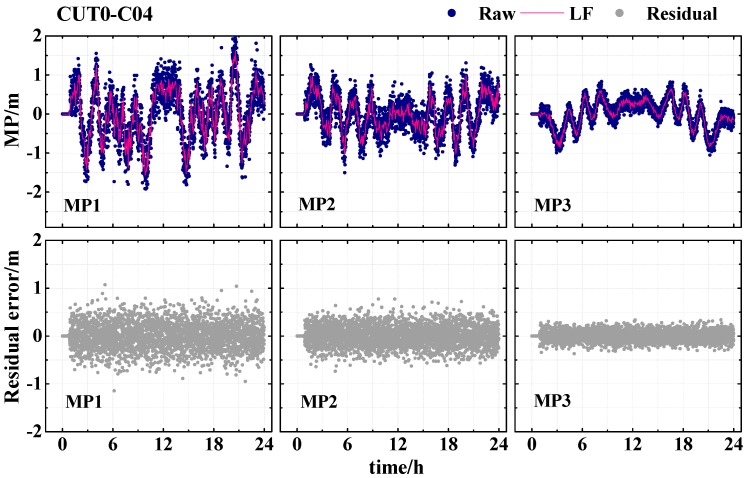
Wavelet analysis results of the C04 satellite at the CUT0 station for DOY 58 in 2017 (27 February 2017). “Raw” represents the original multipath series, “LF” represents the low-frequency component extracted from the multipath series, and “Residual” indicates the multipath residual series after subtracting the low-frequency component.

**Figure 6:**
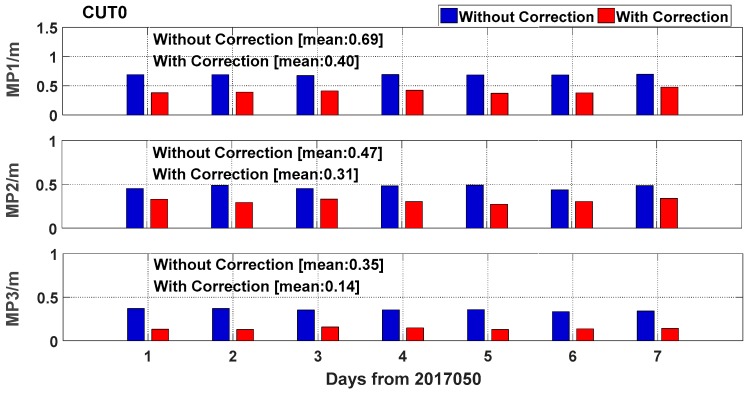
Root mean square error (RMSE) of the one-week multipath series of C04 satellite at CUT0 without (blue) and with (red) multipath correction.

**Figure 7:**
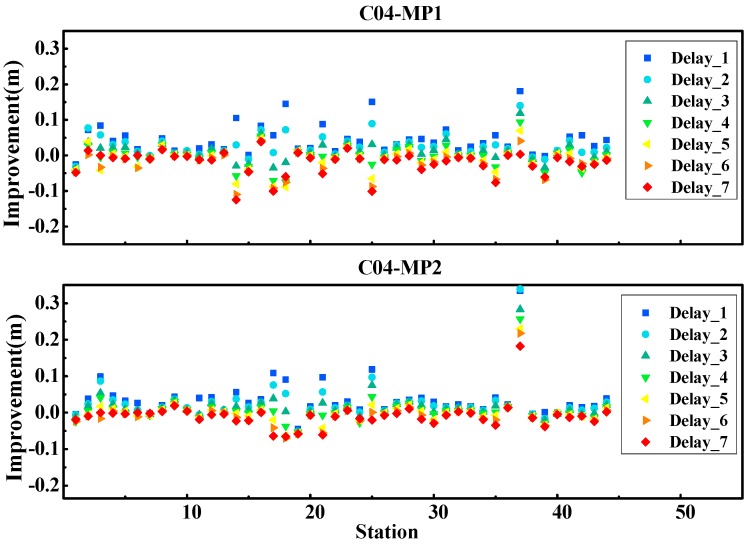
Improvement of the multipath series (RMSE) by correcting the multipath errors with different time delays (from one-day to seven-days).

**Figure 8:**
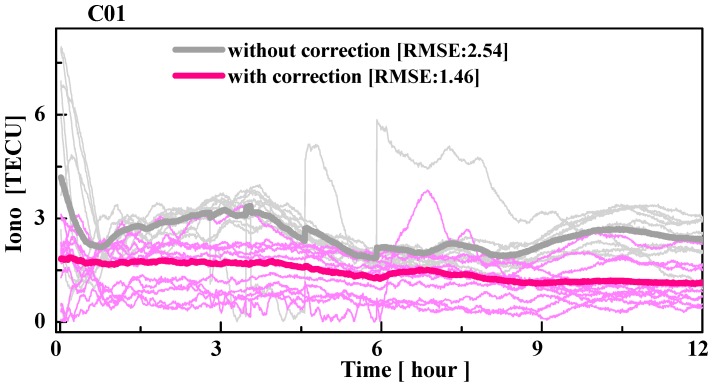
Time series of the ionospheric residual at the CUT0 station of C01 from DOY 46 to 66 in 2017 (16 February 2017 to 7 March 2017). The grey and red lines represent the ionospheric residuals without and with multipath correction, respectively. The thin and bold lines represent each arc and average values, respectively.

**Figure 9:**
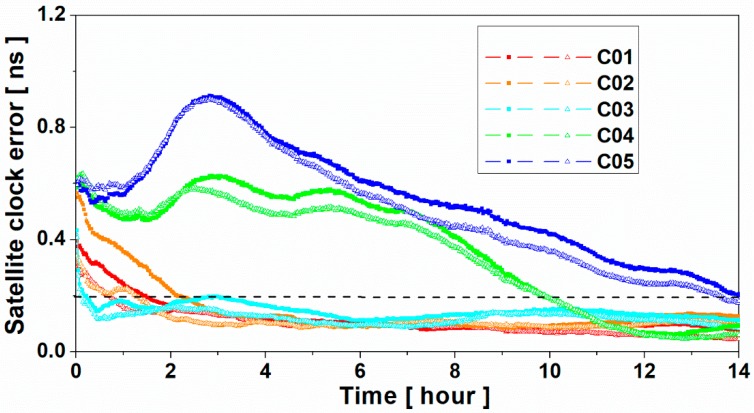
BeiDou Navigation Satellite System (BDS) satellite clock error time series without (line with solid square) and with (line with empty triangle) multipath correction.

**Table 1:** Statistical results of each geostationary orbit (GEO) satellite for all stations without and with correction of multipath error (unit: m).

	Without Correction			With Correction		
	MP1	MP2	MP3	MP1	MP2	MP3
C01	0.24	0.21	0.25	0.20	0.17	0.23
C02	0.24	0.20	0.24	0.19	0.15	0.22
C03	0.19	0.16	0.25	0.16	0.13	0.23
C04	0.25	0.20	0.26	0.20	0.16	0.24
C05	0.20	0.17	0.33	0.16	0.14	0.31
Ave.	0.23	0.19	0.27	0.18	0.15	0.25

**Table 2:** Root mean square error (RMSE) of the ionospheric residual without and with the corrections of multipath error.

	Without Corrections [TECU]	With Corrections [TECU]	Site Number
**C01**	2.05	1.76	43
**C02**	1.86	1.65	32
**C03**	1.08	0.91	29
**C04**	2.82	2.33	24
**C05**	2.35	1.91	19
**Ave.**	2.03	1.71	

## Appendix A

Wavelets are building block functions and are localized in time or space, or both. They are obtained from a single function $\psi (t)\in L^{2}(R)$, called the mother wavelet, by translations and dilations [31], as follows:

(A1) $$\psi_{a,b}(t)=\frac{1}{\sqrt{\left|a\right|}}\psi (\frac{t-b}{a})$$

where ψ(t) is the wavelet basis; *a* represents the dilation parameter; and *b* is the translation parameter, (a,b∈R,a≠0).

In order to satisfy the analysis of the complex signals, the theory of time–frequency transformation is further promoted, and wavelet transform is thus produced. The continuous wavelet transform of a signal *f* is as follows:

(A2) $$W_{f}(a,b)=\frac{1}{\sqrt{a}}\int_{-\infty }^{+\infty }f(t)\bar{\psi (\frac{t-b}{a}})dt$$

where $\bar{\psi }(t)$ is the complex conjugate of ψ(t), and the signal can be reconstructed from the following:

(A3) $$f(t)=\frac{1}{C_{W}}\int_{-\infty }^{+\infty }\int_{-\infty }^{+\infty }\frac{1}{a^{2}}W_{f}(a,b)\psi (\frac{t-b}{a})dadb$$

where the constant satisfies the following admissibility condition:

(A4) $$C_{W}=\int_{-\infty }^{+\infty }\frac{|\hat{\psi }(\omega ){|}^{2}}{\left|\omega \right|}d\omega <\infty$$

where ${\hat{\psi }}_{\left(\omega \right)}$ is the Fourier transform of the mother wavelet ψ(t), and ω is the signal frequency.

In practical applications such as signal processing, a finite number of data points are usually given. A discrete version of the wavelet transform is then required, where discrete dilation and translation parameters are used. When considering the computational efficiency, the dyadic a and b values are used, as follows:

(A5) $$a=2^{j},b=k2^{j}$$

where *j* and *k* are integers. For some particular choices of ψ(t), there exists a corresponding discrete wavelet $\psi_{j,k}(t)$ that has good time–frequency localization properties, such that [40] the following:

(A6) $$\psi_{j,k}(t)=2^{-\frac{j}{2}}\psi (2^{-j}t-k)$$

forms an orthonormal basis for $L^{2}(R)$. Using the orthonormal basis, any $f(t)\in L^{2}(R)$ can be expressed as follows:

(A7) $$f(t)=\sum_{j,k=-\infty }^{+\infty }\alpha_{j,k}\psi_{j,k}(t)$$

where the discrete wavelet coefficient $\alpha_{j,k}$ is defined by the following:

(A8) $$\alpha_{j,k}=\int_{R}f(t){\bar{\psi }}_{j,k}(t)dt$$

The wavelet transform defined by Equations (A5)–(A8) is the discrete dyadic wavelet transform. In this discrete wavelet analysis, a signal can be decomposed into its approximations and details. The detail at level *j* is defined as [21] follows:

(A9) $$D_{j}(t)=\sum_{k\in Z}\alpha_{j,k}\psi_{j,k}(t)$$

where Z is the set of positive intergers. The approximation at level *J* is defined as the sum of the details up to that level, as follows:

(A10) $$A_{J}(t)=\sum_{j>J}D_{j}(t)$$

Then, the signal *f(t)* can be expressed by the following:

(A11) $$f(t)=A_{J}(t)+\sum_{j<J}D_{j}(t)$$

From the formula above, we can know that the approximations are related to one another by the following:

(A12) $$A_{J-1}(t)=A_{J}(t)+D_{J}(t)$$

Equations (A11) and (A12) provide the tree structure of a signal, and also the reconstruction procedure of the original signal. The decomposed approximations and details capture the different frequency bands at different levels, giving information that may not be clearly seen in the original data.
